# Substance use during pregnancy

**DOI:** 10.12688/f1000research.7645.1

**Published:** 2016-05-13

**Authors:** Ariadna Forray

**Affiliations:** 1 Department of Psychiatry, Yale School of Medicine, New Haven, CT, USA

**Keywords:** drug abuse, pregancy, prenatal substance use

## Abstract

Prenatal substance use is a critical public health concern that is linked with several harmful maternal and fetal consequences. The most frequently used substance in pregnancy is tobacco, followed by alcohol, cannabis and other illicit substances. Unfortunately, polysubstance use in pregnancy is common, as well as psychiatric comorbidity, environmental stressors, and limited and disrupted parental care, all of which can compound deleterious maternal and fetal outcomes. There are few existing treatments for prenatal substance use and these mainly comprise behavioral and psychosocial interventions. Contingency management has been shown to be the most efficacious of these. The purpose of this review is to examine the recent literature on the prenatal use of tobacco, alcohol, cannabis, stimulants, and opioids, including the effects of these on maternal and fetal health and the current therapeutic options.

## Introduction

In the United States, women comprise 40% of those with a lifetime drug use disorder and 26% of those who meet criteria for both an alcohol and drug use disorder during the prior 12 months ^1^. Furthermore, women are at highest risk for developing a substance use disorder during their reproductive years (18–44), especially ages 18–29 ^2^. This means that women who are pregnant or soon to become pregnant are at increased risk for substance abuse. According to a national survey conducted in the United States in 2012, 5.9% of pregnant women use illicit drugs, 8.5% drink alcohol and 15.9% smoke cigarettes ^3^, resulting in over 380,000 offspring exposed to illicit substances, over 550,000 exposed to alcohol and over one million exposed to tobacco in utero. Similar patterns of use have been observed in Europe ^4, 5^ and Australia ^6^. The most commonly used substance in pregnancy is nicotine, followed by alcohol, marijuana and cocaine ^7, 8^. However, polysubstance use is as high as 50% in some studies ^7, 9^. Recently, there has been an increase in opiate use in pregnancy. Between 2000 and 2009, the United States saw a five-fold increase in opiate use in pregnancy, coincident with an “epidemic” of opiate prescription misuse ^10– 12^.

There is little information available on the extent of substance use, other than tobacco, among pregnant women in low-income and middle-income countries. The overall prevalence of tobacco use in these countries is 2.6%, with some countries having much higher maternal rates– up to 15% ^13^. While data on illicit substance use in pregnancy is lacking for most middle- and low-income countries, according to the World Health Organization, cannabis is the most common illicit drug worldwide, followed by amphetamine-type stimulants and opiates ^14^, and, as such, they are likely to be used by women of reproductive age. The limited data available for Africa is from South Africa, and indicates that between 3.6 and 8.8% of pregnant women use illicit substances and 19.6% use alcohol ^15^. The most commonly used illicit substances in South Africa include methamphetamine and cannabis ^16^. Opiate use has also increased in places like Africa and Asia ^17^, and is likely to become more prevalent in pregnancy.

Prenatal substance use can bring about several deleterious consequences for both mother and baby, as described in detail below. The concern for the impact of substances on the developing fetus can motivate some women to curb their drug and alcohol use during pregnancy ^18^. In the only prospective study on prenatal substance use, 96% of women with heaving drinking, 78% of women with marijuana use, 73% of women with cocaine use, and 32% of cigarette smokers succeeded in achieving abstinence during pregnancy ^9^. Offsetting the reduction in pregnancy-related use is the dramatic rise in substance use from 6 to 12 months postpartum ^9^. The study showed relapse in 58% of abstinent smokers, 51% of abstinent women who used alcohol, 41% of abstinent women who used marijuana and 27% of abstinent women who used cocaine in the 3 months following delivery ^9^. Thus, while the levels of abstinence in pregnancy may be high, the impact of this is diminished due to the high rates of relapse postpartum. Unfortunately, maternal relapse happens at a time of high childcare needs and when infant development is dependent on maternal bonding. It is also important to note that this was a study conducted in the United States and that the levels of abstinence may not be equivalent in other countries, especially middle- and low-income countries where women may encounter significant socioeconomic stressors, low levels of education, and limited available treatments for substance use.

As evidenced by these data, substance use in pregnancy is still a critical public health concern. The purpose of this review is to provide a brief overview of the pregnancy outcomes, neonatal and long-term developmental consequences of prenatal substance use, and current available treatments for pregnant women.

## Adverse effects of substance use in pregnancy

Heavy alcohol use in pregnancy has been associated with a range of negative birth outcomes, including increased risks of miscarriage ^19^, stillbirth and infant mortality ^20, 21^, congenital anomalies ^22^, low birthweight ^23^, reduced gestational age ^24^, preterm delivery ^25^, and small-for-gestational age ^22, 26, 27^. The evidence for low to moderate alcohol use in pregnancy has either been inconclusive ^28^ or shown no increased risk for these adverse pregnancy outcomes ^29^. Alcohol use in pregnancy has the most well established adverse fetal health effects ^30– 32^ and is associated with the development of fetal alcohol spectrum disorders ^33– 35^ and adverse neurodevelopmental outcomes ^36^. In addition, prenatal drinking is associated with long-term effects, such as cognitive and behavioral challenges ^37, 38^, adverse speech and language outcomes ^39^, executive functioning deficits in children ^40^, and psychosocial consequences in adulthood ^41^.

Smoking during pregnancy exerts direct adverse effects on birth outcomes, including damage to the umbilical cord structure ^42^, miscarriage ^43^, increased risk for ectopic pregnancy ^44^, low birthweight ^45– 47^, placental abruption ^45, 46, 48^, preterm birth ^45, 49^, and increased infant mortality ^45, 46, 48^. Also of concern are the deleterious health effects of second-hand smoke on newborns, which include higher rates of respiratory and ear infections, sudden infant death syndrome, behavioral dysfunction and cognitive impairment ^50^. Additionally, women who were smokers before pregnancy might stop breastfeeding early so that they can take up smoking again ^51^.

Some pregnant women view cannabis use as harmless in pregnancy ^52^; however, it has been linked with several deleterious effects, including preterm labor, low birthweight, small-for-gestational age, and admission to the neonatal intensive care unit ^53^. Prenatal cannabis use has also been linked with adverse consequences for the growth of fetal and adolescent brains ^52^, reduced attention and executive functioning skills, poorer academic achievement and more behavioral problems ^54^. The adverse effects of marijuana are frequently observed with comorbid substance use, and are greatest in heavy users.

The extent of the adverse effects of cocaine use in pregnancy has been overestimated at times. However, there have been several large and thorough studies recently, which have all identified several risk factors associated with cocaine use during pregnancy, including premature rupture of membranes, placental abruption, preterm birth, low birthweight, and small for gestational age infants ^55, 56^. There have been inconsistent reports on the long-term effects of prenatal cocaine exposure on language, motor, and cognitive development, with a few studies describing positive findings ^57, 58^ and some studies reporting very little or no effects ^59^. This inconsistency is probably connected to the confounding effects of the postnatal environment, including unsteady and disordered home environments, dysfunctional parenting, and heavy maternal polysubstance use ^60– 62^. Similar to cocaine use in pregnancy, methamphetamine use is linked with shorter gestational ages, lower birthweight ^63^, fetal loss ^64^, developmental and behavioral defects ^65^, preeclampsia, gestational hypertension, and intrauterine fetal death ^66^.

Opioid use in pregnancy is correlated with a greater risk of low birthweight, respiratory problems, third trimester bleeding, toxemia and mortality ^12, 67^. Maternal opiate use is associated with an increased risk of neonatal abstinence syndrome (NAS), whereby opiate exposure in utero triggers a postnatal withdrawal syndrome ^12^. Anywhere from 45 to 94% of infants exposed to opioids in utero, including methadone and buprenorphine, can be affected by NAS ^12^. NAS results in substantial neonatal morbidity and increased healthcare utilization ^12, 67^, and consists of an array of signs and symptoms, including irritability, feeding difficulties, tremors, hypertonia, emesis, loose stools, seizures, and respiratory distress ^68^. Opioid exposure in pregnancy has also been associated with postnatal growth deficiency, microcephaly, neurobehavioral problems, and sudden infant death syndrome ^67^. Cigarette smoking, which is very common in pregnant women with an opioid use disorder (77–95%) ^69, 70^, may confound the effect of opioid use on poor pregnancy outcomes.

A significant point to take into account is that the undesirable consequences of prenatal substance use are confounded by the frequency of coexisting substance use and comorbid psychiatric illness ^71, 72^. Women with substance use disorders also frequently experience inadequate prenatal care, poor nutrition, chronic medical problems, poverty, and domestic violence ^73, 74^. Furthermore, substance use in pregnancy may also result in an early dysfunctional maternal-infant relationship that can potentiate the negative effects of prenatal drug exposure ^60, 61^.

## Treatment of substance use in pregnancy

There are only a small number of effective therapies for substance use in pregnancy, which primarily involve behavioral counseling (see Table 1). Brief interventions ^75^, in particular those that utilize motivational interviewing ^76, 77^, have been shown to reduce prenatal alcohol use. A recent randomized trial utilizing a telephone-based brief intervention suggests that this method may achieve similar results to the in-person intervention method of moderating prenatal drinking ^78^. Some additional interventions to reduce prenatal drinking that have recently been described include screening via non-healthcare community workers ^79^, counseling by midwives ^80^, and multimedia and educational efforts aimed at improving awareness ^81^.

**Table 1.** Description of behavioral interventions for substance use disorders.

Contingency management (CM)	Based on the principle of positive reinforcement as a means of operant conditioning to influence behavior change. The premise behind CM is to systematically use reinforcement techniques, usually monetary vouchers, to modify behavior in a positive and supportive manner. Originally used for the treatment of cocaine users, it has since been used for opioids, marijuana, cigarettes, alcohol, benzodiazepines, and other drugs.
Motivational interviewing (MI)	A patient-centered, collaborative and highly empathic counselling style for eliciting behavior change by helping clients to explore and resolve ambivalence. It draws from the trans theoretical model of change in order to improve treatment readiness and retention.
Cognitive Behavioral Therapy (CBT)	A psychotherapeutic treatment that uses an easy-to-learn set of strategies to help patients understand the situations that lead them to undesirable thoughts, feelings, or behaviors, to then avoid those situations when possible, and to deal more effectively with such situations when they occur. The goal of these strategies is to break old patterns of responding and replace them with new ones.

As with alcohol, behavioral counseling is the main treatment for smoking cessation and relapse prevention in pregnant women. Unfortunately, psychotherapeutic interventions have had only moderate success ^82– 85^. Pharmacological treatments for smoking cessation have not been evaluated with respect to their safety and efficacy in pregnant and postpartum women ^82, 86^. Randomized clinical trials with nicotine replacement therapy in pregnant women have demonstrated limited efficacy in increasing the rates of abstinence ^87– 90^. The most successful intervention for prenatal smoking cessation is contingency management (CM) with financial incentives ^91– 93^, which has also reportedly improved birth outcomes ^94^.

Treatments specifically aimed at prenatal cannabis use are lacking. The current recommendation for lowering the use of cannabis in pregnancy includes the screening of pregnant women to increase the early identification of cannabis use ^52^. Motivational interviewing (MI) ^95, 96^, cognitive-behavioral therapy (CBT) ^95– 99^, and CM therapies have had some success in reducing marijuana use in women, but they have not been evaluated specifically with pregnant users. Thus, novel interventions that explicitly target cannabis use are vital, particularly given the current tendency towards marijuana legalization.

Existing evidence-based treatments for cocaine use in pregnancy include CBT, MI and CM ^100^. As with smoking, CM is the intervention that shows most potential for treating cocaine-using pregnant women ^62^. A randomized trial found that CM was associated with much longer duration of cocaine abstinence, higher number of cocaine-negative urine tests, and a greater proportion of documented abstinence when compared to community reinforcement approach and twelve-step facilitation ^101^. Currently, there are no evidence-based pharmacological treatments for prenatal cocaine use. Nevertheless, a recent randomized, placebo-controlled trial supports the use of oral micronized progesterone as an intervention for postpartum cocaine use ^102^. The study showed that women randomized to placebo had more self-reported cocaine use compared to women receiving micronized progesterone during the 12 weeks of the trial ^102^. While these are preliminary findings and will require confirmation in a larger clinical trial, they show promise for the application of progesterone in postpartum women to reduce their cocaine use. Treatments for other stimulant use, such as methamphetamine, are limited. Research into reinforcement-based therapy (RBT) combined with a women-focused intervention among pregnant methamphetamine users reported a reduction in methamphetamine use over time ^103^. However, there were no substantial distinctions between the intervention and control conditions ^103^, not unlike another study using RBT to treat stimulant use in pregnancy ^104^. RBT seems to have potential as an intervention for methamphetamine use but more research is required.

Methadone maintenance is the standard care for pregnant women with opiate use disorders ^105^. Conversion from illicit opioid use to opioid maintenance therapy in a medically supervised setting decreases maternal and neonatal morbidity. Methadone maintenance offers greater relapse prevention with a steady opioid dosing regimen, reduces risk-taking behavior, enhances compliance with prenatal care, and leads to better neonatal outcomes ^106^. On the other hand, medication-assisted withdrawal, that is detoxification by gradually reducing the dose of an opioid substitute medication, is associated with a high opioid relapse rate and higher fetal morbidity and mortality rates ^106^. Buprenorphine has recently emerged as another potential therapy for opioid use in pregnancy. A randomized controlled trial that compared methadone and buprenorphine in pregnant opioid users showed that infants whose mothers received buprenorphine needed less treatment for NAS, substantially lower doses of morphine to treat NAS symptoms, and had shorter stays in hospital, compared to the infants of women given methadone ^107^. Notably, buprenorphine had lower retention rates with flexibly delivered doses and low fixed doses compared to methadone ^108^. However, buprenorphine and methadone are equally effective when given as fixed medium or high doses ^108^. CM has likewise been reported to be effective in treating opioid use in pregnancy, by significantly increasing abstinence and treatment attendance compared to controls ^109^. Thus, CM appears to be an important addition to methadone or buprenorphine treatment in pregnant women.

## Breastfeeding and postpartum substance use

Breastfeeding has the potential to be a useful tool for substance use in the postpartum period. Breastfeeding is the only available intervention shown to reduce NAS severity in opioid-exposed newborns ^110, 111^. Breastfeeding might also be protective for postpartum relapse. For example, among breastfeeding smokers, 10% stop breastfeeding because of smoking, and over half of recent or current smokers reported that smoking affected their infant feeding decision ^112^. In addition, non-current smokers are more likely to initiate and continue breastfeeding compared to current smokers ^113, 114^. Therefore, the promotion of breastfeeding might prevent or delay postpartum relapse.

While studies evaluating the potential role of breastfeeding as an intervention for substance use postpartum are limited, the rationale for such interventions is clear. Lactation reduces the HPA response to physical stress ^115^. A behavior that promotes relaxation and reduces stress would be helpful to women with substance use disorders since psychosocial stress increases cravings ^116^. While hormones released during lactation may mediate stress reduction, such hormones have other properties that may help women cope with addiction. Considerable attention has been dedicated to oxytocin, a hormone released during delivery and lactation. Oxytocin administration is under investigation for treatment of drug and alcohol use disorders ^116– 118^. In addition, lactation is positively associated with cognitive and motor development in the infant ^119^. It is well known that stable attachment among children increases resiliency and protects against the development of addiction later in life ^120, 121^. Thus, an intervention that promotes lactation and intimacy through skin-to-skin contact may enhance stable attachment, and have the intergenerational benefit of protecting offspring from the development of addictive and other problematic behaviors ^120, 122, 123^.

## Conclusions

Substance use in pregnancy remains a significant public health problem, which can lead to several harmful maternal and neonatal outcomes. Which drug is being used and the degree of use, as well as the point of exposure, all influence the effects of drug use in pregnancy. In addition to the direct effects of drug exposure in utero, several other variables are associated with deleterious maternal and infant consequences, including psychiatric comorbidity, polysubstance use, limited prenatal care, environmental stressors and disrupted parental care. In conjunction, these factors can negatively influence pregnancy and infant outcomes, and should be taken in to account when developing interventions for prenatal substance use treatments. Many of the health problems associated with substance use in the prenatal period could be avoided given effective and well-timed medical care or intervention. Empirically-driven interventions for prenatal substance are needed. While there are few treatment options for substance use in pregnancy, CM seems to show the greatest promise as an effective therapy for the substances in which it has been studied. Future research needs to focus on developing tailored, safe, and acceptable treatments that can capitalize on pregnancy as a “teachable” moment that can motivate women to adopt risk-reducing health behaviors ^124– 127^.
